# A Non-Invasive Laboratory Panel as a Diagnostic and Prognostic Biomarker for Thrombotic Microangiopathy: Development and Application in a Chinese Cohort Study

**DOI:** 10.1371/journal.pone.0111992

**Published:** 2014-11-05

**Authors:** Tao Zhang, Huimei Chen, Shaoshan Liang, Dacheng Chen, Chunxia Zheng, Caihong Zeng, Haitao Zhang, Zhihong Liu

**Affiliations:** 1 National Clinical Research Center of Kidney Diseases, Jinling Hospital, Nanjing University School of Medicine, Nanjing 210002, P. R. China; 2 Jiangsu Key Laboratory of Molecular Medicine, Nanjing University School of Medicine, Nanjing, 210093, P. R. China; West Virginia University, United States of America

## Abstract

**Background:**

Thrombotic microangiopathy (TMA) in the kidney is a histopathologic lesion that occurs in a number of clinical settings and is often associated with poor renal prognosis. The standard test for the diagnosis of TMA is the renal biopsy; noninvasive parameters such as potential biomarkers have not been developed.

**Methods:**

We analyzed routine parameters in a cohort of 220 patients with suspected TMA and developed a diagnostic laboratory panel by logistic regression. The levels of candidate markers were validated using an independent cohort (n = 46), a cohort of systemic lupus erythematosus (SLE) (n = 157) and an expanded cohort (n = 113), as well as 9 patients with repeat biopsies.

**Results:**

Of the 220 patients in the derivation cohort, 51 patients with biopsy-proven TMA presented with a worse renal prognosis than those with no TMA (P = 0.002). Platelet and L-lactate dehydrogenase (LDH) levels showed an acceptable diagnostic value of TMA (AUC = 0.739 and 0.756, respectively). A panel of 4 variables - creatinine, platelets, ADAMTS13 (a disintegrin and metalloprotease with thrombospondin type 1 repeats 13) activity and LDH - can effectively discriminate patients with TMA (AUC = 0.800). In the validation cohort, the platelet and LDH levels and the 4-variable panel signature robustly distinguished patients with TMA. The discrimination effects of these three markers were confirmed in patients with SLE. Moreover, LDH levels and the 4-variable panel signature also showed discrimination values in an expanded set. Among patients undergoing repeat biopsy, increased LDH levels and panel signatures were associated with TMA status when paired evaluations were performed. Importantly, only the 4-variable panel was an independent prognostic marker for renal outcome (hazard ratio = 3.549; P<0.001).

**Conclusions:**

The noninvasive laboratory diagnostic panel is better for the early detection and prognosis of TMA compared with a single parameter, and may provide a promising biomarker for clinical application.

## Introduction

Thrombotic microangiopathy (TMA) is a pathological lesion that results in thrombosis in capillaries and arterioles, due to an endothelial injury [1]–[3]. TMA lesions in the kidney usually present in two different forms with considerable overlap: (1) glomerular involvement with capillary thrombi, capillary loops with double contours and mesangiolysis with microhemorrhage, that is most frequently seen in patients with hemolytic uremic syndrome; or (2) predominant arteriolar involvement with thrombi and fibrinoid necrosis, particularly in thrombotic thrombocytopenic purpura and malignant hypertension [4], [5].

TMA lesions occur in a number of other kidney diseases as well, including IgA nephropathy, systemic lupus erythematosus (SLE), antiphospholipid antibody syndrome, systemic sclerosis, preeclampsia, infections, medications and post transplantation [2], [6], [7]. The presence of TMA in the kidney has been proven to be associated with poor renal prognosis [8], [9]. Nephrologists often face a typical situation in which patients are suspected of having TMA lesions on the basis of renal disorders. In such cases, it is difficult to decide on an early therapy before the results of a renal biopsy are obtained [10]. While the interpretation of the renal biopsy has become more standardized and the invasive procedure safer over time, bleeding and subsequent functional impairment nevertheless still occur, especially in the patients with coagulopathy [11].

Heterogeneous disorders with TMA are usually characterized by microangiopathic hemolytic anemia (MAHA) [12], thrombocytopenia and/or ischemic organ failure. It is questionable as to whether these abnormalities can predict TMA, since they are also observed in patients without TMA, and studies have been inconsistent [13], [14]. Hence, we enrolled cohorts of patients with renal damage and MAHA and/or thrombocytopenia to determine whether a single parameter or a diagnostic panel could predict the histological TMA. In addition, the association between the noninvasive prediction and a poor renal outcome was further investigated.

## Methods

### Ethics statement

The study was approved by the Ethics Committee of Jinling Hospital. Because this study was retrospective, the Ethics Committee agreed to waive the requirement for the informed consent, and the data were analyzed anonymously.

### Patient selection and study design

We enrolled a cohort of suspected patients from the Research Institute of Nephrology, Jinling Hospital, Nanjing University School of Medicine, PR China during July 2011 to July 2012 (n = 266). The enrollment criteria were: renal damage (proteinuria, hematuria or renal dysfunction) and microangiopathic hemolytic anemia (hemoglobin level below 120 g/L for males, 110 g/L for females, at least 5 schistocytes per high power field in a peripheral blood smear, elevated LDH above 240 u/L [normal 60–240 u/L]) or thrombocytopenia (platelet counts below 100×10^9^/L of blood at any time in the course of the disease) [15]. Patients were randomly divided into two cohorts: (1) derivation cohort (n = 220) and (2) validation cohort (n = 46).

In addition, 157 patients diagnosed with SLE were selected from the suspected patients, and 113 patients were enrolled in an expanded group for external validation. SLE was defined according to the 1997 American College of Rheumatology revised criteria for SLE [16]. The independent patients were enrolled in the expanded group according to the criteria: (1) renal damage (proteinuria, hematuria or renal dysfunction) and (2) fever (temperature of >38°C, no infection), elevated LDH (more than 240 u/L) or non-renal anemia. The information and profiles of patients during the follow-up sessions were also reviewed through October 2013.

### Definitions

TMA in the kidney was defined by the histologic feature of occlusive fibrin-platelet thrombi in at least one glomerulus or one arteriole, with one or more of the following: (1) glomerular endothelial swelling and detachment, capillary wall thickening and double contour formation, mesangial lysis with microhemorrhage, and erythrocytolysis, and/or (2) obliterative arteriolopathy defined as luminal occlusion with mural myxoid or fibrinoid change, thickening of the vessel wall, with or without erythrocytolysis, luminal thrombosis and concentric spindle cell proliferation or hypercellularity [2], [5].

All patients accepted adjunctive treatments, including protecting the organ function, symptomatic and immunosuppressive treatment. Glomerularfiltration rate was estimated (eGFR) using the simplified MDRD (Modification of Diet in Renal Disease) formula. End stage renal disease (ESRD) was defined as eGFR<15 mL/min/1.73 m^2^ or a need for permanent dialysis therapy. During follow-up, the combined event defined as ESRD or a doubling of the serum creatinine level and death. The renal survival rate was defined as the percentage of patients who had preservation of renal function independent of ESRD and death, while the survival rate was defined as the percentage of live patients.

### Laboratory features

Physical exams and routine laboratory tests were performed on the suspected patients. Serum ADAMTS13 activity was measured by the Fluorescence Resonance Energy Transfer (FRET) assay (United States Patent No.7270976) [17]. Serum anti-ADAMTS13 IgG antibodies (Sekisui Diagnostics, USA), vWF (von Willebrand factor) (Sunbiote, Shanghai, China), thrombomodulin (Diaclone Research, Besancon, France), E-selectin (R&D Systems, Minneapolis, Minnesota, USA) and soluble vascular cellular adhesion molecule-1 (sVCAM-1, VCAM) (R&D Systems, Minneapolis, Minnesota, USA) were measured using enzyme linked immunosorbent assays. The concentrations of endothelial cells in the circulation were sorted using a magnetic microbead sorting system (MACS, Miltenyi Biotec, Germany), according to the manufacturer’s instructions [18].

### Statistical analysis

All data were given a numerical code and statistical analysis was performed using SPSS software, version 19.0 or RMS software, version 2.12.2. Comparisons of proportions or mean values between patients with or without TMA in the kidney were calculated by the Mann-Whitney test or chi-square (χ2) test.

Logistic regression was used to identify 6 parameters - hemoglobin, platelets, serum creatinine, LDH, ADAMTS13 activity and THBD - that discriminated between patients with and without TMA in the kidney. Regression estimated from this panel defined a diagnostic signature or individual predictors, and the greatest area under the receiver-operating-characteristic (ROC) curve as the best-fitting model was used. The area under the curve (AUC) was calculated, and sensitivity and specificity were used to evaluate the ability of these to discriminate TMA in the kidney. The best sub-set panel was then fit to 1000 additional bootstrap samples. Cross-validated measures of discrimination (i.e., the AUC), model fit (i.e., calibration-curve intercept and slope) and a locally estimated scatterplot-smoothed (loess) calibration plot were obtained [19].

Cumulative incidence of poor renal outcome was calculated using Kaplan-Meier survival probabilities (1- survival probabilities) and comparisons were made using the log-rank test. Cox regression was performed to test the association of the pathologic findings and the 4-variable panel signature with the renal outcome. A two-tailed P value less than 0.05 is considered to be statistically significant.

## Results

**Figure 1:**
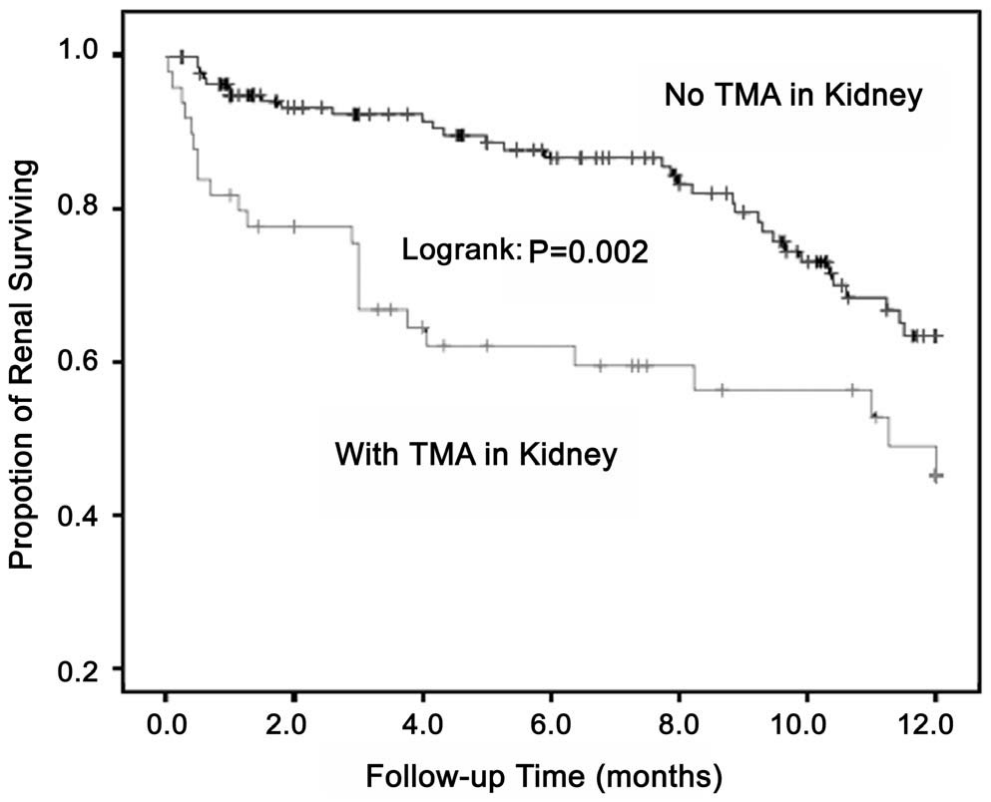
Comparison of renal outcome in suspected patients. After 12 months of follow-up, the patients with TMA had a worse outcome of renal survival than those without TMA (Log rank: P = 0.002).

In the derivation cohort of 220 patients, 51 (23.2%) patients were histologically proven to have TMA by renal biopsy. Compared with patients without TMA, the patients with TMA presented a worse renal outcome (P = 0.002, log-rank test; Figure 1) at the 12-month follow-up, with a renal survival rate of 54.9% (P = 0.015; Table 1).

**Table 1:** Clinical laboratory data for suspected patients in derivation cohort (n = 220).

	TMA	No TMA	P Value
**Number of patients**	**51**	**169**	
Age (year)	26 (963)	28 (1370)	0.450
Male (n, %)	10 (19.6)	45 (26.6)	0.310
**Clinical diagnosis**			
HUS/TTP (n, %)	15 (29.4)	0 (0)	<0.001
Primary glomerulonephritis & acute interstitial nephritis (n, %)	0 (0)	22 (13.0)	0.003
Autoimmune diseases (n, %)	29 (56.9)	144 (85.2)	<0.001
SLE (n, %)	27/29 (93.1)	130/144 (90.3)	1.000
Pregnancy/postpartum (n, %)	5 (9.8)	1 (0.6)	0.003
Malignant hypertension (n, %)	1 (2.0)	0 (0)	0.232
Post-transplantation (n, %)	1 (2.0)	2 (1.2)	0.549
**Laboratory profiles**			
Urine protein (g/24 h)	1.5 (0.310.6)	2.5 (0.29.8)	0.073
Erythrocyturia (×10^4^/mL)	18 (110000)	24 (19000)	0.364
Hemoglobin (g/L)	74.2±16.1	87.1±21.6	<0.001
Platelets (×10^9^/L)	73 (10275)	108 (34441)	<0.001
Serum creatinine (umol/L)	347.2 (52.21697.5)	162.7 (33.61556.0)	<0.001
Globulin (g/L)	22.0±5.7	23.3±6.6	0.289
C3 (g/L)	0.62±0.29	0.57±0.30	0.219
C4 (g/L)	0.16±0.10	0.14±0.10	0.098
L-lactate dehydrogenase (u/L)	418 (1292920)	267 (64878)	<0.001
ADAMTS13 antibody (Au/mL)	16.6 (2.184.9)	16.6 (1.681.3)	0.862
ADAMTS13 activity (ng/mL)	616±331	772±255	0.001
NEC (/ml)	19.7±6.8	20.7±8.9	0.654
E-selectin (ng/mL)	65.2±37.3	73.1±72.8	0.636
VCAM (ng/mL)	2011±1070	2460±1340	0.073
Thrombomodulin (ng/mL)	6.3±4.3	4.4±2.8	<0.001
vWF (%)	184±127	215±120	0.080
**After 12-months of follow up**			
Renal survival rate (%)	54.9 (28/51)	74.6 (126/169)	0.015
Survival rate (%)	94.1 (48/51)	98.2 (166/169)	0.138

Values are expressed as medians (range), means ± standard deviation or percentages. *P* values were calculated by Mann-Whitney U test, Fisher’s exact test or chi-square test as appropriate. HUS: hemolytic uremic syndrome; TTP: thrombotic thrombocytopenic purpura; SLE: systemic lupus erythematosus; C3: Complement component 3; C4: Complement component 4; ADAMTS13: A Disintegrin and Metalloprotease with ThromboSpondin type 1 repeats 13; NEC: normal endothelial cells; VCAM: vascular cell adhesion molecule; vWF: von Willebrand factor.

### Correlation between laboratory parameters and TMA lesions

**Figure 2:**
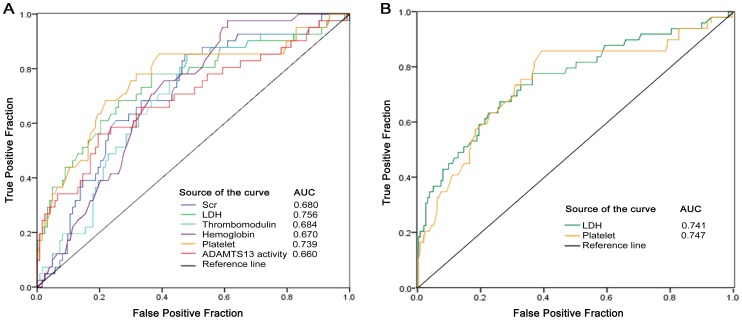
Receiver operating characteristic curves of laboratory parameters. (A) The fraction of true positive results (sensitivity) and the fraction of false positive results (1-specificity) for LDH, HGB, SCr, PLT, THBD and ADAMTS13 activity were developed in 220 patients (all P≤0.001), and the levels of platelet and LDH showed acceptable discrimination, with AUC 0.739 and 0.756, respectively. (B) The levels of platelet and LDH could discriminate patients with TMA from those with no TMA in the validation cohort (n = 46), with AUC 0.747 and 0.741, respectively.

The levels of serum creatinine, LDH and thrombomodulin (THBD) were significantly higher in patients with TMA than those without TMA, while levels of hemoglobin, platelets and ADAMTS13 activity were remarkably lower (all P<0.05; Table 1). With a cutoff of 0.05 (P value, in Table 1), 6 laboratory parameters were included in the next binary logistic regression analysis. The ROC curve showed that these 6 parameters individually can distinguish patients with TMA in the derivation set (all P<0.05; Figure 2A). Only the platelet and LDH levels showed acceptable discrimination values (0.7≤AUC<0.8), while levels of hemoglobin, serum creatinine, ADAMSTS13 activity and THBD had low discrimination accuracy (0.5≤AUC<0.7). With the use of the cutoff point of 97.5×10^9^/L, platelets had 82.4% sensitivity and 60.9% specificity, and with the cutoff point of 289 u/L, LDH had 67.3% sensitivity and 74.1% specificity (Table S1 in File S1).

In an independent validation cohort, platelet and LDH levels also could discriminate patients with TMA from those without TMA (Figure 2B; Table S2 in File S1). Thus, the levels of platelets and LDH showed acceptable predictive probabilities of patients with TMA in the kidney.

### Correlation between the 4-variable panel and TMA lesions

Furthermore, multiple logistic regression analysis in the derivation set indicated that levels of serum creatinine (SCr), platelets (PLT), LDH and ADAMTS13 activity were valid predictors of renal TMA lesions (all P<0.05; Table 2), with the final panel signature:

**Table 2:** Results of logistic regression analysis (n = 220).

Variable	B	S.E	Sig.	Exp (B)	95% CI
					Lower-Upper
Serum creatinine	0.140	0.060	0.020	1.150	1.022–1.295
LDH	0.004	0.001	0.002	1.004	1.001–1.006
Platelet	−0.010	0.004	0.021	0.990	0.981–0.998
ADAMTS 13 Activity	−0.002	0.001	0.014	0.998	0.996–1.000

Abbreviations: B: coefficient of regression; S.E.: Standard Error; Sig: P value; Exp (B): odds ratio; CI: confidence interval. LDH: L-lactate dehydrogenase; ADAMTS 13: a disintegrin and metalloprotease with thrombospondin type 1 repeats 13.



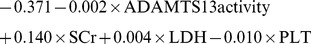



The units of measurement are as listed in Table 1. In the equation, −0.371 was the intercept, and −0.002, 0.140, 0.004 and −0.010 were the slopes (coefficients), respectively, for the ADAMTS13 activity, SCr (mg/dL), LDH and PLT values in the best-fitting logistic-regression model. The intercept and slopes have no intrinsic units of measurement.

**Figure 3:**
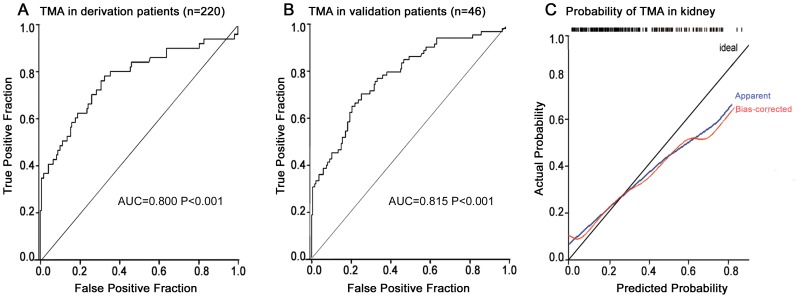
Receiver operating characteristic curves and calibration curve for a diagnostic panel. (A) The diagnostic panel was developed in the derivation group of 220 suspected patients, with AUC 0.800, P<0.001. (B) This marker was validated in 46 independent patients, with AUC 0.815, P<0.001. (C) Bootstrap validation shows the calibration curve of the diagnostic panel. Cross-validated estimates of the AUC, calibration-curve intercept and slope were 0.777, 0.07 and 0.64, respectively. The loess-smoothed estimates of the cross-validated and unadjusted calibration curves are overlaid on a diagonal reference line representing good model calibration.

The ROC curve showed that this 4-variable model yielded an AUC of 0.800 (95% confidence interval [CI], 0.723 to 0.877; P<0.001), suggesting a good discrimination between patients with TMA in the kidney and those without TMA (Figure 3A). With the use of the cutoff point of 0.248, this diagnostic panel has 81.6% sensitivity and 66.9% specificity. The diagnostic panel for the prediction of TMA was validated in the independent cohort, with an AUC of 0.815 (P<0.001; Figure 3B). The use of the cut-off of 0.248 predicted the presence of TMA with 80.0% sensitivity and 61.5% specificity.

Bootstrap validation of this 4-variable panel yielded a cross-validated estimate of the AUC of 0.777, which is an estimate of the expected value of the AUC in the combined derivation and validation cohorts. The calibration-curve intercept and slope were 0.07 and 0.64, respectively. It was revealed that the predicted probabilities of a biopsy showing TMA in the kidney tended to be relatively higher than the actual probabilities (Figure 3C). The loess-smoothed estimates of the unadjusted and cross-validated calibration curves were overlaid on a diagonal reference line representing good model calibration (P = 0.489). The close correspondence of the two curves to the reference line shows good fit and reflects the above interpretation of the intercept and slope estimates of the calibration curve (Figure 3C).

### Evaluation of LDH, platelet and the 4- variable panel in extra cohorts

**Figure 4:**
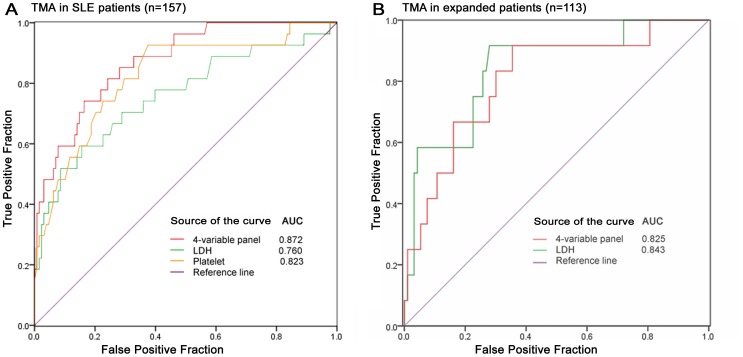
Evaluation of LDH, platelet and 4-variable panel in extra cohorts. (A) All levels of platelet, LDH and the 4-variable panel were evaluated in 157 suspected patients diagnosed with SLE and could discriminate patients with TMA, with an AUC of 0.823, 0.76 and 0.872, respectively. (B) To further validate in another independent group of 113 patients and ROC curve analysis showed that the levels of LDH and the 4-variable panel yielded an AUC of 0.843 and 0.825, showing an good discrimination of TMA (both P<0.001).

Since renal TMA lesions occur in a number of kidney diseases, we focused on a specific condition and 157 renal patients diagnosed with SLE were selected in extra validation (Table S3 in File S1). All levels of platelets, LDH and the 4-variable panel could discriminate patients with TMA in SLE. The ability of discrimination in the 4-variable panel was higher than the levels of platelets and LDH, with an AUC of 0.872 (Figure 4A). With the use of the cutoff point of 0.248, the diagnostic panel has 74.1% sensitivity and 83.6% specificity.

To further enhance the application of these markers, we enrolled another independent group of 113 patients and determined the association between the levels of LDH, platelets and the 4-variable panel and TMA lesions (Table S4 in File S1). ROC curve analysis showed that the levels of LDH and the 4-variable panel yielded an AUC of 0.843 and 0.825, showing a good discrimination of TMA (both P<0.001; Figure 4B). However, platelet levels could not predict patients with TMA lesions in this cohort (P = 0.068).

### Evaluation of LDH and the 4-variable panel in patients with repeat biopsy

**Figure 5:**
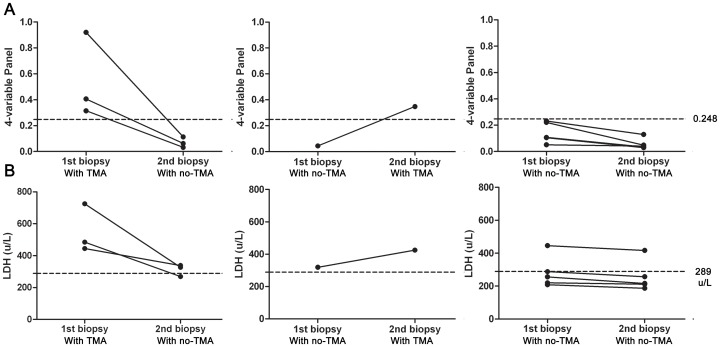
Evaluation of LDH and 4-variable panel in patients with repeat biopsy. (A)We analyzed paired first and second levels of LDH and 4-variable panel in a subset of 9 patients, who underwent repeat renal biopsy. The first figure shows 4-variable panel signature plummeted from 0.248 or greater in the same subset of patients, when three patients presented TMA at first biopsy but no TMA at second biopsy. In the second figure, only one patient had no TMA at first biopsy and TMA at second biopsy and 4-variable panel dramatically increased to 0.348. For other 5 patients, 4-variable signature was significantly decreased after treatment, although they consistently presented without TMA at two biopsies and the signature was 0.248 or less. (B) Similar pattern of LDH changes was observed in the patients with repeat biopsy, and increased LDH levels were associated with TMA status.

We then analyzed, in a subset of 9 patients who underwent repeat renal biopsy, levels of LDH and the 4-variable panel in paired blood samples taken at the time of the first and second biopsy (Table S5 in File S1). It is interesting to note that the 4-variable panel signature plummeted from 0.248 or higher in the same subset of patients when they presented with TMA at the first biopsy, but with no TMA at second biopsy (Figure 5A). On the contrary, for the only patient who had no TMA at first biopsy but exhibited TMA at the second biopsy, the 4-variable panel dramatically increased to greater than 0.248. For the other 5 patients, the 4-variable signature was significantly decreased after treatment (P = 0.030), although they each presented without TMA at the two biopsies and the signature was 0.248 or less.

A similar pattern of LDH changes were observed in the patients with repeat biopsy, and increased LDH levels were associated with TMA status (Figure 5B). However, an LDH value of 289 u/L was not a good cut-off level to discriminate TMA status in this group of patients.

### Association of LDH levels and the 4-variable panel with renal survival

**Figure 6:**
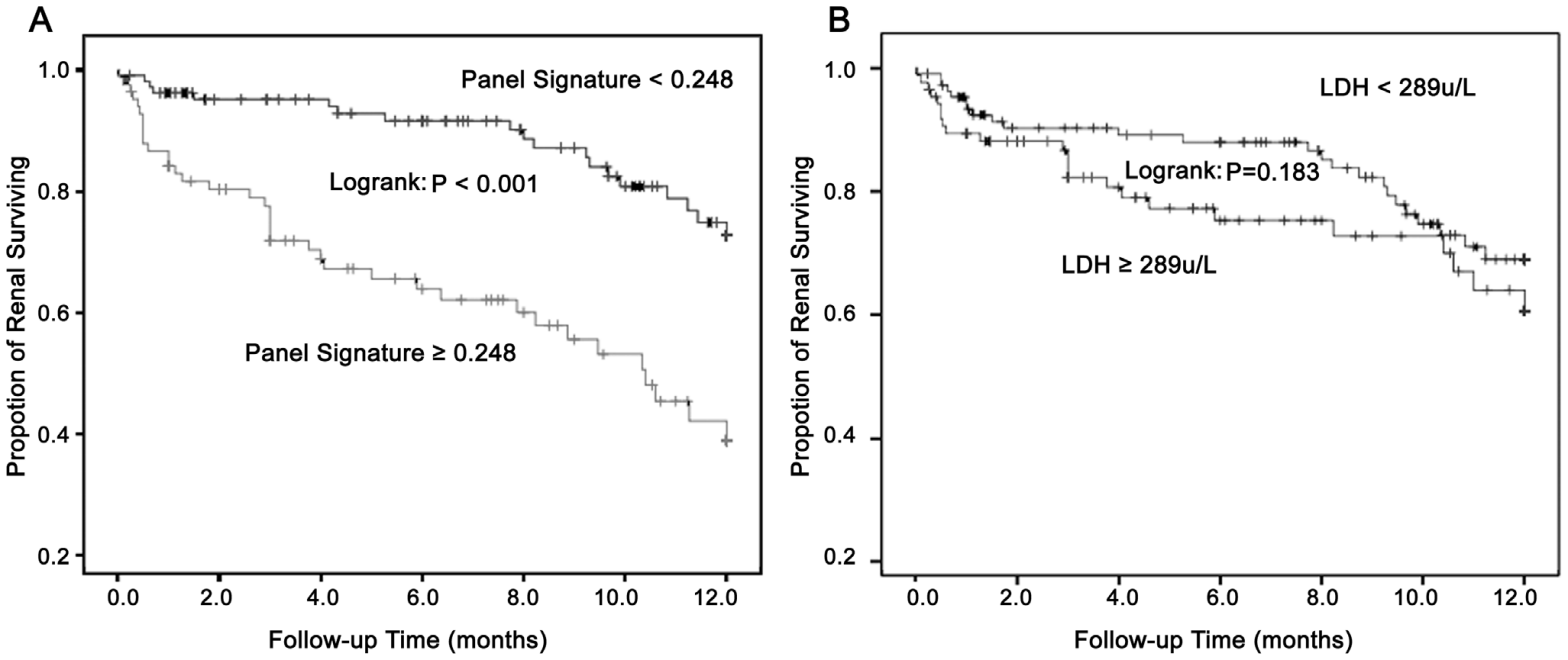
Association of LDH levels and 4-variable panel with renal survival. (A) Using the cutoff value of 0.248, suspected patients were divided into two groups by more or less than the value and the renal survival of two groups followed up 12 months was significantly different (P<0.001). (B) The pattern of increased LDH concentrations (more than the cutoff point of 289 u/L) associated statistically poorer renal outcome was not observed (P = 0.183).

To further evaluate whether LDH levels and the 4-variable panel can serve as a predictor of renal survival in patients with suspected TMA, we performed a Kaplan-Meier survival analysis. As suspected, patients with a higher 4-variable panel signature had a statistically significant worse renal outcome (P<0.001, log-rank test; Figure 6A). However, the pattern of increased LDH concentrations was not associated with a poorer renal outcome (P = 0.183, log-rank test; Figure 6B).

Furthermore, the renal survival was only 53.9% in patients with a panel signature of more than 0.248, but was 79.5% in patients with a panel signature of less than 0.248. The Cox proportional hazard survival regression model revealed that TMA lesions increased the risk for a poor renal outcome, with a hazard ratio of 2.235 (95% CI 1.306 to 3.826, P = 0.003), while a panel signature 0.248 or greater had a hazard ratio of 3.549 (95% CI 2.034 to 3.549, P<0.001). These two diagnostic factors showed a similar predictive effect on renal outcome (P = 0.964).

## Discussion

Histological TMA in the kidney has been frequently described in association with a large number of underlying diseases [20]–[22]. Although the causes of TMA in the kidney are unclear, it has been reported that there is a significantly worse renal outcome among patients with TMA [16], [22]. Kaplan et al. [23] reported that death rates were as high as 25% and progression to ESRD occurred in half of the patients with TMA within 10 years after diagnosis. In agreement with published studies [24], we confirmed that patients with renal biopsy-proven TMA presented with a poor outcome in the kidney and the patients with TMA had a 2.24-fold higher risk of renal failure than those without TMA.

The renal biopsy represents the gold standard in the management of patients with TMA, but a noninvasive diagnostic method for detection of TMA would be a valuable clinical application. Few studies have focused on the prediction of the histopathologic lesions, although abnormalities in the urinalysis and an increase in serum creatinine have been observed in patients with TMA lesions [25]. In addition, reports have demonstrated that laboratory variables were involved in clinical conditions associated with TMA [26]–[28], including hemolytic uremic syndrome and thrombotic thrombocytopenic purpura. Increased levels of LDH are associated with the severity of hemolysis and tissue ischemia, which might increase the risk of tissue lesions [29]. A deficiency of ADAMTS13 was related to thrombus formation and was subsequently noted in patients with thrombocytopenic purpura, who often suffered from TMA lesions in the kidney [30], [31].

This study is the first to demonstrate the potential of routine laboratory parameters to be used in the detection of TMA without renal biopsy. Six parameters - serum creatinine, platelets, hemoglobin, THBD, ADAMTS13 activity and LDH - had moderate diagnostic value for TMA. The strengths of this study are that we developed a diagnostic panel based on 4 laboratory variables (levels of serum creatinine, LDH, platelets and ADAMTS13 activity). This panel can noninvasively and accurately predict histological TMA. This is supported by the high AUC values of 0.800 derived from patients with and without TMA (sensitivity 81.6%; specificity 66.9%). Similar effects were noted when the predictive panel was validated using an independent population. The cross-validation further revealed that the 4-variable model tended to have high sensitivity, but a relatively low specificity. To the best of our knowledge, this is the first noninvasive biomarker that can detect TMA in the kidney. In addition, each individual parameter of the 5 or 6 variable model could not reach statistical significance (P<0.05, data not shown). The model of fewer variables could discriminate patients with TMA in derivation cohort, including the levels of LDH and platelets, but this effect was not proven by all different validation cohorts.

The 3 markers, LDH, platelets and the 4-variable panel, were further evaluated in a group of patients with SLE and expanded sets of patients. SLE is often reported with TMA lesions in the kidney [6], [22], and there were 157 patients diagnosed with SLE among the suspected patients investigated. Focusing on one particular disease, all 3 markers showed discrimination values for patients with TMA. Because some patients with TMA exhibit little or no signs of clinical manifestations [31]–[33], we expanded the application of these markers. LDH levels and the 4-variable panel still demonstrated good discrimination, while platelets did not. Thus, LDH levels and the 4-variable panel might have wide usage in different cohorts of patients. In addition, data from patients with a repeat biopsy confirmed the association of increased LDH levels and the 4-variable signature with TMA status.

Another important finding of our study was that the 4-variable panel also serves as a prognostic biomarker for renal patients. The increased signature of the 4-variable panel was an independent prognostic parameter. The prognostic value of the 4-variable panel was similar with the histological diagnosis of TMA. As a noninvasive biomarker, the 4-variable panel showed an advantage in clinical application. However, LDH levels did not exhibit a prognostic value for renal outcome. Therefore, the 4-variable panel might not only diagnose TMA but also help predict renal outcome with a higher accuracy than other single laboratory parameter.

Although our current assay may become a promising diagnostic tool for TMA, we acknowledge three potential limitations of this study. It is retrospective and will need to be validated with a prospective cohort of patients. Furthermore, the evaluation of prognosis was rendered difficult because of the variable nature of the treatments received. In addition, the number of patients with certain diseases was small and some validations were calculated using non-independent data, which prevented an in-depth evaluation of the usefulness of the diagnostic panel for specifically predicting TMA in the kidney.

## Conclusions

In conclusion, our results provide compelling evidence for the potential use of laboratory parameters as a noninvasive diagnostic and prognostic tool for TMA in the kidney. The 4-variable panel showed an advantage for the early detection of renal TMA lesions without renal biopsy and in directly predicting renal prognosis. In order for this concept to be incorporated into routine clinical practice in the near-future, validation is needed in large-scale prospective trials.

## Supporting Information

File S1 Includes table S1–S5. **Table S1,** Diagnosis of TMA in kidney among patients (n = 220). **Table S2,** Laboratory variables in the validation group of internal patients (n = 46). **Table S3,** Clinical laboratory data for suspected patients with SLE (n = 157). **Table S4,** Clinical laboratory data for patients in expanded group (n = 113). **Table S5,** The laboratory feature of patients with/without TMA in the kidney at first renal biopsy and repeat biopsy.
(DOCX)
