# Supplementary material for: A Non-Invasive Laboratory Panel as a Diagnostic and Prognostic Biomarker for Thrombotic Microangiopathy: Development and Application in a Chinese Cohort Study
Source: PLoS One. 2014 Nov 5;9(11):e111992. doi: 10.1371/journal.pone.0111992 (PMC4221199; doi:10.1371/journal.pone.0111992)
Supplement: File S1 — Includes table S1–S5. Table S1, Diagnosis of TMA in kidney among patients (n = 220). Table S2, Laboratory variables in the validation group of internal patients (n = 46). Table S3, Clinical laboratory data for suspected patients with SLE (n = 157). Table S4, Clinical laboratory data for patients in expanded group (n = 113). Table S5, The laboratory feature of patients with/without TMA in the kidney at first renal biopsy and repeat biopsy. (DOCX) [file pone.0111992.s001.docx]

**Supplementary material**

Supplement to: Tao Zhang. et al., Non-invasive prediction of TMA

**Index**

Table S1 page 2

Table S2 page 3

Table S3 page 4

Table S4 page 5

Table S5 page 6

**Table S1.** **Diagnosis of TMA in kidney among patients (n=220).**

| Predictor Variables | Area under the ROC Curve #  (95% confidence interval) | P value | Sensitivity* | Specificity |
| --- | --- | --- | --- | --- |
| Hemoglobin | 0.670 (0.589-0.750) | <0.001 | 0.608 | 0.657 |
| Platelet | 0.739 (0.654-0.823) | <0.001 | 0.824 | 0.609 |
| Serum Creatinine | 0.680 (0.601-0.759) | <0.001 | 0.824 | 0.515 |
| LDH | 0.756 (0.673-0.840) | <0.001 | 0.673 | 0.741 |
| ADAMTS13 activity | 0.660 (0.567-0.754) | 0.001 | 0.549 | 0. 775 |
| Thrombomodulin | 0.684 (0.594-0.774) | <0.001 | 0.857 | 0.516 |

# The area under the ROC (receiver operating characteristic curve) ranges between 0-1

* Clinical relevant point on ROC with sensitivity of at least 70%.

TMA: Thrombotic microangiopathy; LDH: L-lactate dehydrogenase; ADAMTS 13: a disintegrin and metalloprotease with thrombospondin type 1 repeats 13.

**Table S2. Laboratory variables in the validation group of internal patients (n=46).**

|  | **With TMA** | **No TMA** | ***P* Value** |
| --- | --- | --- | --- |
| Number of patients | 20 | 26 |  |
| Platelet (×10^9^/L) | 75.1±56.6 | 137.3±87.1 | 0.004 |
| Serum Creatinine (umol/L) | 307.7±281.1 | 199.8±167.1 | 0.240 |
| L-lactatedehydrogenase (u/L) | 483.1±245.3 | 313.1±186.0 | 0.001 |
| ADAMTS13 activity (ng/mL) | 651.5±304.7 | 769.0±259.8 | 0.400 |
| Value of panel | 0.428±0.270 | 0.185±0.144 | 0.001 |

Values are expressed asmeans ±standard deviation. *P*values were calculated byMann–Whitney U test as appropriate. TMA: thrombotic microangiopathy; LDH: L-lactate dehydrogenase; ADAMTS 13: a disintegrin and metalloprotease with thrombospondin type 1 repeats 13.

**Table S3. Clinicallaboratory data for suspected patients with SLE (n=157).**

|  | **With TMA** | **No TMA** | **P Value** |
| --- | --- | --- | --- |
| **Number of patients** | **27** | **130** |  |
| Age (year) | 26 (13～61) | 27 (14～64) | 0.575 |
| Male (n, %) | 3 (11.1) | 26 (20.0) | 0.414 |
| **Laboratory profiles** |  |  |  |
| Urine protein (g/24h) | 1.7 (0.2～7.4) | 2.7 (0.2～9.8) | 0.047 |
| Erythrocyturia(×10^4^/mL) | 26 (1～10000) | 22 (1～9000) | 0.713 |
| Hemoglobin (g/L) | 74.1 ± 14.1 | 85.0 ± 19.0 | 0.005 |
| Platelets (×10^9^/L) | 63 (10～211) | 108 (27～346) | <0.001 |
| Serum Creatinine (umol/L) | 259.9 (54.8～1005.2) | 136.2 (33.6～1004.3) | <0.001 |
| Globulin (g/L) | 22.5 ± 6.4 | 23.0 ± 6.6 | 0.902 |
| C3 (g/L) | 0.47 ± 0.18 | 0.46 ± 0.22 | 0.465 |
| C4 (g/L) | 0.11 ± 0.06 | 0.11 ± 0.07 | 0.420 |
| L-lactate dehydrogenase (u/L) | 487 (129～1189) | 272 (64～794) | <0.001 |
| ADAMTS13 antibody (Au/mL) | 16.6 (2.1～65.6) | 16.6 (3.0～81.3) | 0.953 |
| ADAMTS13 activity (ng/mL) | 530.8 ± 310.9 | 767.9 ± 250.0 | <0.001 |
| NEC (/ml) | 18.6 ± 7.1 | 21.1 ± 9.0 | 0.228 |
| E-selectin (ng/mL) | 56.1 ± 29.2 | 66.6 ± 69.0 | 0.839 |
| VCAM (ng/mL) | 2264 ±1271 | 2640 ±1350 | 0.208 |
| Thrombomodulin (ng/mL) | 7.2 ± 5.2 | 4.1 ± 2.8 | <0.001 |
| vWF (%) | 195 ± 145 | 223 ± 122 | 0.132 |
| **After 12-months of follow up** |  |  |  |
| Renal survival rate (%) | 59.3 (16/27) | 78.5 (102/130) | 0.036 |
| Survival rate (%) | 96.3 (26/27) | 98.5 (128/130) | 0.424 |

Values are expressed as medians (range), means± standard deviation or percentages. *P* values were calculated by Mann–Whitney U test or Fisher’s exact test as appropriate.SLE: systemic lupus erythematosus; TMA: Thrombotic microangiopathy; C3: Complement component 3; C4: Complement component 4; ADAMTS13: A Disintegrin and Metalloprotease with ThromboSpondin type 1 repeats 13; NEC: normal endothelial cells; VCAM: vascular cell adhesion molecule; vWF: von Willebrand factor.

**Table S4. Clinical laboratory data for patients in expanded group (n=113).**

|  | **With TMA** | **No TMA** | **P Value** |
| --- | --- | --- | --- |
| **Number of patients** | **13** | **100** |  |
| Age (year) | 33 (12～62) | 31 (13～76) | 0.791 |
| Male(n, %) | 2 (15) | 36 (36) | 0.213 |
| **Clinical Diagnosis** |  |  |  |
| HUS/TTP | 2 (15) | 0 (0) | 0.012 |
| Primary glomerulonephritis&acute interstitial nephritis(n, %) | 0 (0) | 46 (46) | 0.001 |
| Autoimmune diseases (n, %) | 9 (69) | 50 (50) | 0.244 |
| SLE (n, %) | 8/9 (89) | 46/50 (92) | 0.577 |
| Pregnancy/postpartum(n, %) | 0 (0) | 2 (2) | 1.000 |
| Malignant hypertension(n, %) | 2 (15) | 2 (2) | 0.065 |
| **Laboratory profiles** |  |  |  |
| Urine protein (g/24h) | 1.4 (0.3～4.2) | 3.2 (0.2～13.9) | 0.014 |
| Erythrocyturia(×10^4^/mL) | 83 (1～1200) | 38 (1～40000) | 0.699 |
| Hemoglobin (g/L) | 93.4 ± 18.2 | 108.7 ± 31.8 | 0.045 |
| Platelets (×10^9^/L) | 188 (101～288) | 208 (107～437) | 0.099 |
| Serum Creatinine (umol/L) | 346.6 (121.1～917.7) | 96.4 (42.4～750.6) | <0.001 |
| Globulin (g/L) | 23.3 ± 6.54 | 21.2 ± 6.8 | 0.204 |
| C3 (g/L) | 0.70 ± 0.28 | 0.79 ± 0.41 | 0.526 |
| C4 (g/L) | 0.16 ± 0.10 | 0.17 ± 0.10 | 0.732 |
| L-lactate dehydrogenase (u/L) | 452 (208～922) | 267 (55～810) | <0.001 |
| ADAMTS13 antibody (Au/mL) | 20.1 (10.3～60.0) | 14.1 (2.3～72.1) | 0.436 |
| ADAMTS13 activity (ng/mL) | 842.1 ± 213.7 | 800.3 ± 218.7 | 0.431 |
| NEC (/ml) | 15.5 ± 7.3 | 13.7 ± 6.8 | 0.498 |
| E-selectin (ng/mL) | 222.3 ± 520.4 | 57.7 ± 37.9 | 0.125 |
| VCAM (ng/mL) | 2312 ±1710 | 2272 ±1280 | 0.645 |
| Thrombomodulin (ng/mL) | 3.8 ± 2.5 | 2.7 ± 1.9 | 0.225 |
| vWF (%) | 350 ± 432 | 215 ± 120 | 0.645 |
| **After 12-months of follow up** |  |  |  |
| Renal survival rate (%) | 61.5 (8/13) | 88.0 (88/100) | 0.012 |
| Survival rate (%) | 92.3 (12/13) | 100.0 (100/100) | 0.115 |

Values are expressed as medians (range), means ± standard deviation or percentages. *P* values were calculated by Mann–Whitney U test or Fisher’s exact test as appropriate. HUS: hemolytic uremic syndrome; TTP: thrombotic thrombocytopenic purpura;SLE: systemic lupus erythematosus; C3: Complement component 3; C4: Complement component 4; ADAMTS13: A Disintegrin and Metalloprotease with ThromboSpondin type 1 repeats 13; NEC: normal endothelial cells; VCAM: vascular cell adhesion molecule; vWF: von Willebrand factor.

**Table S5. The laboratory feature of patients with/without TMA in the kidney at first renal biopsy and repeat biopsy.**

| Pt  no | | Age, y | Gender | | Clinical diagnosis | | **First biopsy** | | | | | | | | |  | | **Repeat biopsy** | | | | | | |
| --- | --- | --- | --- | --- | --- | --- | --- | --- | --- | --- | --- | --- | --- | --- | --- | --- | --- | --- | --- | --- | --- | --- | --- | --- |
|  |  |  |  |  |  |  | Platelets  ×10^9^/L | | Creatinine umol/L | | LDH  u/L | ADAMTS13activity, ng/mL | | | Signature of panel |  | | Platelets  ×10^9^/L | | Creatinine umol/L | | LDH  u/L | ADAMTS13activity, ng/mL | Signature of panel |
| **First renal biopsy with TMA and repeat renal biopsy without TMA** | | | | | | | | | | | | | | | | | | | | | | | | |
| 1 | 16 | | | F | SLE | 96 | | 217.5 | | 444 | | | 584.6 | 0.406 | | | | 246 | 435.9 | | 338 | | 959.9 | 0.062 |
| 2 | 16 | | | M | SLE | 112 | | 101.7 | | 484 | | | 691.1 | 0.315 | | | | 226 | 77.8 | | 269 | | 994.5 | 0.032 |
| 3 | 26 | | | M | SLE | 30 | | 150.2 | | 725 | | | 15.0 | 0.920 | | | | 153 | 122.0 | | 327 | | 828.2 | 0.113 |
| **First renal biopsy without TMA and repeat renal biopsy with TMA** | | | | | | | | | | | | | | | | | | | | | | | | |
| 1 | 15 | | | F | SLE | 212 | | 107.9 | | 319 | | | 1020.6 | 0.044 | | | | 78 | 164.4 | | 425 | | 717.6 | 0.348 |
| **First renal biopsy and repeat biopsy both Without TMA** | | | | | | | | | | | | | | | | | | | | | | | | |
| 1 | 34 | | | M | SLE | 80 | | 83.1 | | 256 | | 1370.8 | | | 0.051 | | 210 | | 74.3 | | 216 | | 911.7 | 0.041 |
| 2 | | 30 | | F | SLE | 79 | | 433.2 | | 209 | | 810.6 | | | 0.221 | | 143 | | 181.2 | | 187 | | 1096 | 0.049 |
| 3 | | 23 | | F | SLE | 133 | | 276.7 | | 446 | | 862.6 | | | 0.231 | | 218 | | 475.6 | | 417 | | 890.3 | 0.129 |
| 4 | | 22 | | F | SLE | 106 | | 41.6 | | 220 | | 816.5 | | | 0.107 | | 218 | | 49.5 | | 212 | | 860.8 | 0.034 |
| 5 | | 30 | | F | SLE | 124 | | 111.4 | | 288 | | 932.4 | | | 0.105 | | 244 | | 76.0 | | 257 | | 923.1 | 0.029 |

Abbreviations: TMA: thrombotic microangiopathy; Pt indicates patient; LDH: L-lactate dehydrogenase; ADAMTS13: A Disintegrin and Metalloprotease with ThromboSpondin type 1 repeats 13; F: female; M: male.SLE: systemic lupus erythematosus.

窗体顶端

|  |
| --- |
